# Towards a rational approach to promoter engineering: understanding the complexity of transcription initiation in prokaryotes

**DOI:** 10.1093/femsre/fuae004

**Published:** 2024-02-21

**Authors:** Cara Deal, Lien De Wannemaeker, Marjan De Mey

**Affiliations:** Centre for Synthetic Biology, Ghent University. Coupure Links 653, BE-9000 Ghent, Belgium; Centre for Synthetic Biology, Ghent University. Coupure Links 653, BE-9000 Ghent, Belgium; Centre for Synthetic Biology, Ghent University. Coupure Links 653, BE-9000 Ghent, Belgium

**Keywords:** transcription initiation, promoter sequence, promoter engineering, sigma factors, synthetic biology

## Abstract

Promoter sequences are important genetic control elements. Through their interaction with RNA polymerase they determine transcription strength and specificity, thereby regulating the first step in gene expression. Consequently, they can be targeted as elements to control predictability and tuneability of a genetic circuit, which is essential in applications such as the development of robust microbial cell factories. This review considers the promoter elements implicated in the three stages of transcription initiation, detailing the complex interplay of sequence-specific interactions that are involved, and highlighting that DNA sequence features beyond the core promoter elements work in a combinatorial manner to determine transcriptional strength. In particular, we emphasize that, aside from promoter recognition, transcription initiation is also defined by the kinetics of open complex formation and promoter escape, which are also known to be highly sequence specific. Significantly, we focus on how insights into these interactions can be manipulated to lay the foundation for a more rational approach to promoter engineering.

## Teaser

Unravelling the intricate and sequence-specific regulation of transcription initiation by promoter sequences is crucial, as an in-depth understanding of promoter–RNA polymerase interactions can pave the way to a more rational approach to promoter engineering.

## Introduction

Synthetic biology aims to apply engineering concepts to biology. In this context, the gold standard of synthetic biology is collections of well-defined, predictable, and tuneable parts that form the building blocks for expression of individual genes and ultimately complex genetic circuits (Arkin 2013, Garcia and Trinh 2019). In the context of industrial biotechnology, the development of toolboxes of predictable and tuneable gene expression parts that function in industrially relevant hosts will be essential for creation of robust and cost-effective microbial cell factories (Lucks et al. 2008, An and Chin 2009, Mutalik et al. 2013, Liu et al. 2018, Costello and Badran 2021).

To achieve predictability, it is important to be able to reduce both environmental and genetic context that can affect the performance of genetic parts. Extensive part characterization and predictive models can help us to predict how environmental factors and surrounding DNA context can alter the regulation of gene expression. In addition, gene regulation that is orthogonal to the host metabolism facilitates predictability by reducing unwanted environmental and host-related context, allowing the part to function largely independently of host stress or environmental changes. Equally, optimizing metabolic flux of an expression pathway is required to prevent negative consequences of overproduction and maximize desired product formation. To ensure optimal expression of each pathway component, a toolbox of genetic control elements is necessary for fine-tuning gene expression levels.

In the context of industrial bioprocesses, it is often beneficial to regulate gene expression at a transcriptional level. As the first step in the gene expression process, this has multiple benefits including conserving energy and cellular resources such as RNA polymerases, and providing the capability to rapidly respond to changes in environmental conditions (Segall‐Shapiro et al. 2014, Bervoets and Charlier 2019, Ma et al. 2022). There are three main stages of transcription: initiation, elongation, and termination. In this review, we focus on transcription initiation as it is the point where transcription specificity is defined, yielding orthogonality (Browning and Busby 2004). Promoter sequences are regions of DNA that, through recognition and interaction with the sigma subunit of RNA polymerase (RNAP), signal the starting point for transcription. Promoter strength is known to vary considerably, meaning that production of full-length transcripts can vary over 10 000-fold for different promoter sequences, making them good targets for regulating gene expression levels (McClure et al. 1983, Mazumder and Kapanidis 2019).

Although extensive collections of functional promoters exist, there is a considerable lack of unique and well-characterized promoters that can be used in industrially relevant hosts (Hossain et al. 2020, De Wannemaeker et al. 2022). In addition, many commonly used promoters are context specific due to their interaction with the host metabolism, resulting in environmental interference, which is highly undesirable when designing robust microbial cell factories that will be subject to harsh environmental conditions (Collado-Vides et al. 1991, Lu et al. 2009, Gilman and Love 2016).

To combat this, well-defined, orthogonal, and tuneable promoters that function in a broad range of host organisms can be created using protein engineering. Despite the fact that protein engineering has already been frequently and effectively utilized to generate variation in gene expression levels, the field can still be improved to further increase the level of control over gene regulation (Blazeck and Alper 2013, Xu et al. 2019). Within promoter sequences there are multiple conserved elements that are known to contribute to successful transcription initiation. Many previous promoter engineering attempts focus on one of these promoter regions at a time, ignoring the complexity of the interactions that define transcriptional output, and therefore limiting the power and predictability of these promoter engineering attempts. The fact that transcription initiation is defined by more than recognition of the promoter sequence by the sigma factor is often overlooked, further compounding the problem. Single mutations, even in the sequence immediately upstream of the transcription start site or upstream of the main promoter boxes can cause global changes in promoter activity, highlighting the complexity of the system and knowledge required when engineering promoters based solely on a small number of promoter elements (Urtecho et al. 2019, Saecker et al. 2021).

Through a more complete understanding of the interactions between the sigma factor and promoter sequence that define the strength and specificity of transcription initiation, we can lay the foundations for a more rational approach to promoter engineering. To this end, several recent studies have further elucidated the complexity of these interactions through development of thermodynamic models that leverage an in-depth knowledge on transcription mechanism to achieve improved predictions of gene expression levels. In addition, the use of massively parallel reporter assays have allowed high throughput assembly and measurement of large promoter libraries that represent different combinations of promoter elements, including upstream and downstream regions, and their interactions with each other, providing large datasets that are already being utilized for forward promoter engineering (Einav and Phillips 2019, Urtecho et al. 2019, Lagator et al. 2020, LaFleur et al. 2022). Such knowledge can greatly expand the potential of the field of promoter engineering through, e.g. creation of smarter promoter libraries that increase tunability, design of promoters with defined regulation or expression properties, or addition of improved insulator sequences that reduce the influence of the surrounding promoter context. Of particular interest is the potential of re-engineering the interaction between the sigma factor and DNA, which could allow the design of promoters with specific desired characteristics and orthogonal interaction with a coengineered sigma factor, allowing gene expression that is primarily controlled independently of the host metabolism.

In the subsequent sections, we will break down the steps involved in transcription initiation and the promoter sequence features that determine the strength and specificity at each stage, demonstrating the complexity of transcriptional regulation by promoter sequences and providing insight into the relative importance of different promoter positions on the process. The information given relates primarily to interactions with *Escherichia coli* σ^70^ although, due to the fact that the mechanism of transcription is highly conserved, much of the information is relevant to promoter interactions with alternative sigma factors and those from other organisms. However, it should be noted that, when considering those sigma factors that differ more significantly in structure and mechanism from σ^70^, there can be significantly different modes of transcriptional regulation than described here. Whilst existing reviews in this area focus on promoter recognition, they do not take into account open complex formation and promoter escape, which are also mediated largely by interactions between promoter and sigma factor and have a significant role in determining transcriptional output (Hook-Barnard and Hinton 2007). Here, we will build on these reviews, highlighting that the kinetics of open complex formation and promoter escape are also highly promoter sequence dependent.

## Regulation of transcription initiation in prokaryotes

**Figure 1.**
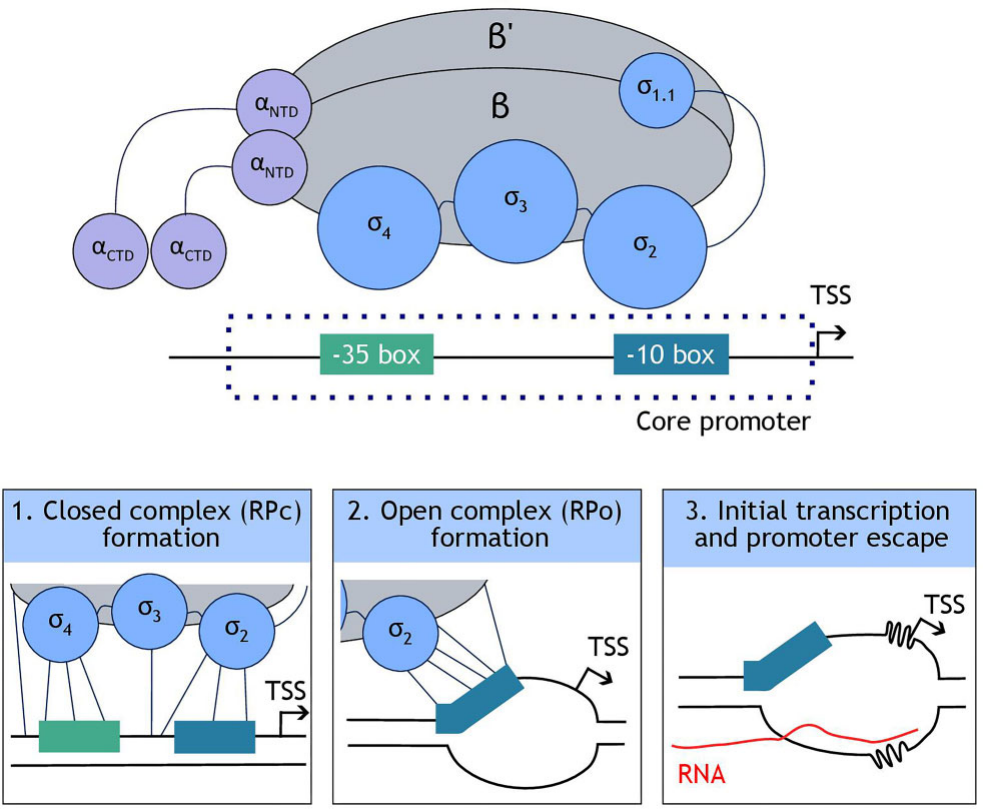
The stages of transcription initiation in prokaryotes. An overview of the interactions between RNAP holoenzyme (ααββ'ωσ) and promoter elements at each stage of transcription is given. TSS denotes the transcription start site.

The steps in transcription initiation are controlled by the interaction of the sigma factor with the promoter sequence and are summarized in Fig. 1. In the light of recent structural studies (Chakraborty et al. 2012, Feklistov et al. 2017, Boyaci et al. 2019, Chen et al. 2020, 2021, Saecker et al. 2021) that have further elucidated the complex and dynamic mechanism and regulation involved in transcription initiation, we have more insight into the role of particular promoter sequence characteristics in determining transcriptional output. However, it should be noted that the specific details of these mechanisms, intermediates and kinetics presented here are still debated and likely differ depending on the promoter sequence, further adding to the complexity (Mazumder and Kapanidis 2019).

In the first step of transcription initiation, the sigma factor (comprising four domains denoted σ_1_, σ_2_, σ_3_, and σ_4_) recognizes the promoter sequence primarily at the conserved −35 and −10 hexamer positions, causing the RNAP holoenzyme (consisting of subunits α, α, β, β', ω, and σ) to be recruited to the promoter. Both specific and nonspecific interactions occur between the promoter and RNAP holoenzyme, forming the closed complex (Fig. 1, box 1). Secondly, promoter melting and open complex formation take place through separation of 13 base pairs of double stranded DNA from position −11 to +2, forming the transcription bubble (Fig. 1, box 2). Initial RNA synthesis and promoter escape is the final stage of transcription initiation and culminates in dissociation of the RNAP holoenzyme from the promoter sequence, allowing it to proceed along the DNA and enter transcription elongation (Fig. 1, box 3). Each of these stages and the specific and nonspecific interactions involved will be described in more detail in the subsequent sections.

### Closed complex formation

As the first step of transcription initiation, initial promoter recognition is of great interest in promoter engineering, as altering the early interaction of the sigma factor with the DNA will directly affect the specificity and strength of a given promoter. In the first stage of promoter recognition RNAP makes contacts with the promoter sequence, forming a closed complex where RNAP directly contacts the double-stranded DNA and spans from positions −55 to +15, relative to the transcription start site (Li and McClure 1998). The closed complex DNA is thought to be bent 17° at the −10 box, positioning downstream promoter DNA above the RNAP DNA binding cleft in preparation for further steps of transcription (Chen et al. 2020). Bending of the −35 box between positions −38 and −48 has also been reported and is also thought to direct the closed complex towards the enzyme active site (Ruff et al. 2015).

A recent study by Feklistov et al. (2017) proposed that fluctuations in RNAP clamp opening and closing allow efficient and dynamic promoter recognition (Feklistov et al. 2017). In this process, the open clamp form of RNAP scans the genome for upstream promoter elements such as the −35 box and UP element. Dynamic binding of RNAP to these sequence features forms the closed complex in which both general and base-specific contacts between upstream promoter elements and the RNAP holoenzyme are formed and further stabilized.

**Figure 2.**
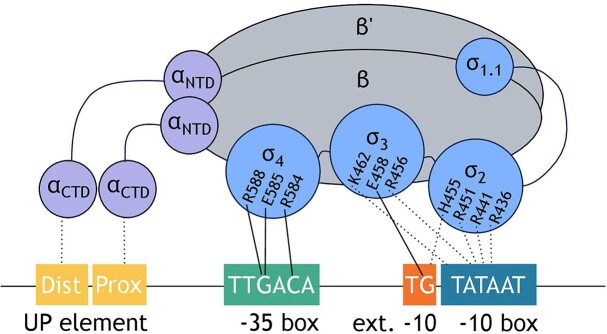
Interactions involved in formation of the closed complex. Both base-specific (solid lines) and nonspecific (broken lines) interactions between RNAP holoenzyme (ααββ'ωσ) and promoter DNA are shown.

However, within closed complex formation there is an abundance of built-in complexity to consider. The dynamic nature of the process means that interactions are readily reversible and are subject to much regulation, limiting the impact of promoter sequence alone in determining transcription rate (Saecker et al. 2021). The relative contributions of conserved upstream elements on the initiation and kinetics of closed complex formation differs between promoters. This is outlined in detail below and summarized in Fig. 2.

#### –35 box interactions

Initial chemical cross-linking studies gave the first indication of both nonspecific and base-pair specific recognition of the −35 box by domain 4 of σ^70^ (Simpson 1979, Park et al. 1980, Hilton and Whiteley 1985, Buckle et al. 1991). Subsequently, amino acid substitutions in the housekeeping sigma factor that alter or relax −35 consensus were identified, implicating positions −33 and −31 in base-specific promoter recognition through interaction with amino acids E585, R588 and R584, respectively (Gardella et al. 1989, Siegele et al. 1989). These studies were complimented by detailed information from crystal structures, which confirmed the base-specific interaction between −33G and −31C of a σ^70^ consensus hexamer with amino acids E585 and R584 that are found in the helix-turn-helix motif of σ_4.2_. In addition, a number of amino acids were identified as having a role in creating nonspecific contacts with −35 box DNA (Campbell et al. 2002, Murakami et al. 2002, Feklistov and Darst 2011, Bae et al. 2015).

Whilst the −35 box consensus is defined as TTGACA, there are numerous hexamer compositions that result in stable interactions and favourable transcription initiation. This is demonstrated by a recent free energy model developed by LaFleur et al. (2022) that provides interaction free energies for different promoter sequence compositions, allowing us to quantify the effect of different promoter positions on transcriptional output. The learned interaction energies indicate that whilst −33G is present in 6 out of 10 of the most favourable hexamers, A, T, and C at this position also feature in the top 10 most favourable −35 box compositions. This data set provides a highly useful resource for determining the stability of interactions between RNAP and DNA at different promoter compositions and can, therefore, be utilized in a rational and data-driven approach to promoter engineering (LaFleur et al. 2022). In addition, a recent study by Liu et al. (2022) has further defined the role and impact of certain positions of the −35 box on closed complex formation through re-engineering the interaction between the −35 box and the sigma factor, giving a further example of how this knowledge can be used in a promoter engineering context to yield promoter sequences that function orthogonally to the host metabolism.

Whilst the sequence of the −35 box is the most conserved of the upstream elements and, at many promoters, has the biggest influence on promoter recognition by the RNAP holoenzyme, this often leads to an oversimplification and assumption that this acts independently to define initial promoter recognition. It is not uncommon for promoters to lack a −35 box, which is compensated for by the presence of other upstream promoter elements, as discussed further below.

#### –10 box interactions

Whilst it was previously suggested that the −10 box was also involved in closed-complex formation, it is now thought that this initial scanning does not recognize specific bases of the −10 box. However, at this stage, DNA-backbone interactions of the −10 box with basic residues of σ_2_ (R436/R441/R451) and σ_3_ (K462/R456) help to stabilize the closed complex and, therefore, influence the productivity of the closed complex (Feklistov et al. 2017, Chen et al. 2020). Additionally, a recent study demonstrated that −35 and −10 box binding are not independent of each other, indicating that the composition of the −10 box influences −35 box recognition (Einav and Phillips 2019). In terms of base-specific interactions, it is likely that a dynamic mechanism exists where −10 box recognition is coupled to DNA melting and is, therefore, discussed further in the context of open complex formation.

#### Extended −10 box interactions

A conserved extended −10 motif, located two base pairs upstream of the −10 box at some promoters, is known to contribute to promoter sequence recognition through interaction with σ_3_. The base at position −14 of the nontemplate strand forms base-specific contacts with amino acid E458, which is located in the α-helix of σ_3_. Amino acid residue H455 of σ_2_ is also thought to make direct contacts with the phosphate backbone of the nontemplate strand at this position, indicating that σ_2_ also has a role in extended −10 box recognition (Barne et al. 1997, Murakami et al. 2002). Whilst the consensus −10 box motif is thought to be TGN, calculated interaction free energy of RNAP–DNA binding indicated that there are multiple compositions that would be favourable for transcription, indicating that this preference is not fixed (LaFleur et al. 2022).

The importance of the extended −10 box in closed complex formation is further demonstrated by its ability to compensate for the absence of other consensus promoter elements. Previous research shows that, in cases where a consensus −35 box is absent, the extended −10 box can act alone to anchor RNAP to the correct position on the DNA (Keiltys and Rosenberg 1987, Barne et al. 1997, Bown et al. 1999, Mitchell et al. 2003, Sanderson et al. 2003, Haugen et al. 2008, Ruff et al. 2015). In addition, the extended −10 box can also compensate for a longer spacer, with a high percentage of promoters containing the extended −10 box consensus ‘TGN’ motif having an 18 bp spacer in comparison to the consensus length of 17 bps (Mitchell et al. 2003, Ruff et al. 2015). Furthermore, the presence of an extended −10 box in combination with a consensus −35 box has been known to compensate for a weak −10 box, further demonstrating the flexibility in the sequence elements involved in promoter recognition (Hook-Barnard and Hinton 2007).

Whilst the extended −10 box is generally less imperative for functional transcription than the −35 or −10 hexamer, the importance of the extended −10 box on transcription is promoter dependent. In one recent study, removing the consensus extended −10 box resulted in a deleterious phenotype, indicating that this motif is required for functionality at certain promoters (Park and Wang 2021). This information indicates that the extended −10 box is an important target for promoter engineering and can be directly modified to alter promoter recognition preference. Further, its ability to compensate for other promoter elements should be considered when designing the promoter engineering strategy, to ensure that the changes made to other promoter elements are not undermined by this compensation.

#### UP element interactions

Some promoters have an AT rich region located upstream of the −35 box that is also implicated in promoter recognition. This sequence, located between −40 and −60 and known as the UP element, helps to anchor the sigma factor at the correct location on the DNA and is also known to cause bending of DNA between −35 and −60, which is thought to stabilize the closed complex and facilitate progression to further steps of transcription (Davis et al. 2011). The C-terminal domain of the two α-subunits of RNAP holoenzyme (α-CTD) interacts with the UP element through both general and base specific interactions (Ross et al. 1993, Estrem et al. 1999, Gourse et al. 2000, Ross et al. 2001). This interaction is sequence dependent as the narrow minor grove characteristic of UP element sequences is known to favour α-CTD domain binding, a trend also observed in the recent free energy model described by Salis and colleagues (Ross et al. 1993, LaFleur et al. 2022). The UP element is composed of two AT rich subsites known as the proximal and distal sites, each of which interacts with one αCTD domain. Promoters may have one or both of these, and it is thought that their contributions to transcriptional output are additive to each other (Einav and Phillips 2019, LaFleur et al. 2022). Whilst the distal element has previously shown to function comparably to the full-length UP element, indicating that it has the greatest influence on promoter recognition (Estrem et al. 1999), more recent studies have shown that both elements can be influential to promoter functionality (LaFleur et al. 2022).

When combined with a canonical −35 box, a consensus UP element sequence was shown to increase transcription up to 330-fold, confirming its potential as an interesting target in the field of promoter engineering (Rao et al. 1994, Estrem et al. 1998, LaFleur et al. 2022). Mutation of the AT rich tracts at consensus UP elements can alter formation and stability of the closed complex, allowing modification of promoter specificity or strength. Previous studies show that DNA bending can be abolished with only single base pair mutations, highlighting the sequence specific nature of the UP element and the potential for altering these interactions in a rational and directed way (Ruff et al. 2015). Like with the extended −10 box, examples of weak −35 elements being compensated for by strong UP elements are abundant, bringing the same caution when considering the rational promoter engineering strategy and showing that, when determining the kinetics and stability of closed complex formation, it is important to consider the additive strength of the combination of promoter elements.

#### Spacer region

The base pairs between the −35 and −10 boxes, known as the spacer region, are also an important determinant of promoter strength and specificity. Correct spacer length is essential to achieve promoter recognition as this anchors the RNAP in position on the DNA so that the required sigma subunits line up with their corresponding promoter elements. A previous study calculated that 44% of *E. coli* promoters have a spacer length of 17 bps (Mitchell et al. 2003). It is well-documented that promoters with this consensus length have higher transcriptional output than identical promoters with either 16 or 18 bp spacer (Stefano and Gralla 1982, Aoyama et al. 1983, Mulligan et al. 1985). To compliment this, a recent RNAP thermodynamic model predicted that interaction free energy of a 16 or 17 bp spacer was most favourable, whilst spacers of length 15, 19, and 20 bp had high positive interaction free energies, indicating that these were considerably less favourable for transcription initiation. Notably, a spacer length of 20 bp gave the most positive interaction free energy of all the promoter compositions in the study, indicating that long spacer length was highly detrimental to functional transcription (LaFleur et al. 2022). Together these demonstrate that spacer length is a highly important determinant of transcriptional output, indicating that altering spacer length is a powerful way to manipulate both promoter strength and specificity.

However, as is the common theme of these promoter recognition interactions, there are examples where nonoptimal spacer length is compensated for. For example, as previously mentioned, consensus extended −10 boxes of housekeeping sigma factors can compensate for longer spacer length, giving functionality to the promoter and further highlighting the need to consider promoter elements in combination when determining the transcriptional output of a given promoter sequence (Mitchell et al. 2003). It is thought that rotation of σ_4_ is required to accommodate different spacer lengths, meaning that spacer length variation is limited by the possible rotation of σ_4_, which is sigma factor dependent (Zuo and Steitz 2015). Interestingly, in the case of σ^S^, amino acid residue E458 binds to spacer region DNA, initiating the formation of a kink in the DNA, which allows this alternative sigma factor to utilize promoters with nonoptimal spacer lengths (Typas and Hengge 2006). This knowledge gives an insight of how sigma factor engineering could be used alongside promoter design to functionalize promoter sequences that have nonoptimal promoter features. Such designed promoter sequences would be nonfunctional for transcription by native sigma factors but functional with a coengineered sigma factor, creating possibilities for orthogonal gene expression.

Aside from spacer length, spacer sequence is also implicated in determining transcription initiation rate. Individual base substitutions in the spacer region have been shown to effect transcription rate at given promoter sequences (Chan and Busby 1989, Mellies et al. 1994, Thouvenot et al. 2004). Consequently, the spacer region is often a target for promoter libraries that aim to modulate promoter strength without influencing specificity (De Mey et al. 2007, Bervoets et al. 2018, Van Brempt et al. 2020). It is likely that spacer sequence influences transcription initiation primarily through changes in DNA secondary structure and curveability (Kanhere and Bansal 2005, Bansal et al. 2014). This can explain the observed influence of GC rich sequences that have previously been shown to be associated with increased promoter activity, compared to AT rich spacers at given promoters (Repoila and Gottesman 2003, Liu et al. 2004, Klein et al. 2021). A recent AI-based tool developed by Wang and colleagues uses a data-driven approach to create promoter regions to flank the −35 and −10 box that are optimized for DNA shape and curvability, giving an example of how this knowledge can be manipulated for a more rational approach to promoter engineering (Zhang et al. 2023).

Interestingly, sequence-specific recognition of the spacer sequence has also been observed in a study by Zenkin and colleagues. A region of the spacer from −22 to −18, with a loosely defined consensus sequence of AACCT, was shown to interact with the β′ subunit of RNAP, facilitating closed complex formation. A truncated promoter, lacking a −35 box and other upstream features was able to initiate transcription in the presence of the Z element, whilst transcription was abolished when the Z element was removed. Furthermore, in the background of a weak −10 box, a consensus Z element worked together with an intact −35 box to allow promoter utilization. This again demonstrates the high level of complexity in the regulation of promoter recognition and the vast array of element combinations that can work together to achieve closed complex formation. It is thought that promoter sequence-specific interactions contribute to this regulation, as the influence of the Z element differed with sequence. Although the exact mechanism of Z element recognition is unknown, it has been linked to conserved residues Y47 and R48 of the β′ subunit, which are in close proximity to the promoter DNA. Mutation of these amino acids abolished Z element interactions, indicating direct contact with the DNA and suggesting they could be manipulated to regulate promoter recognition preferences (Yuzenkova et al. 2011). These insights can further guide promoter engineering strategies that target the spacer region, potentially increasing the level of promoter tuneability that can be achieved.

### Open complex formation

**Figure 3.**
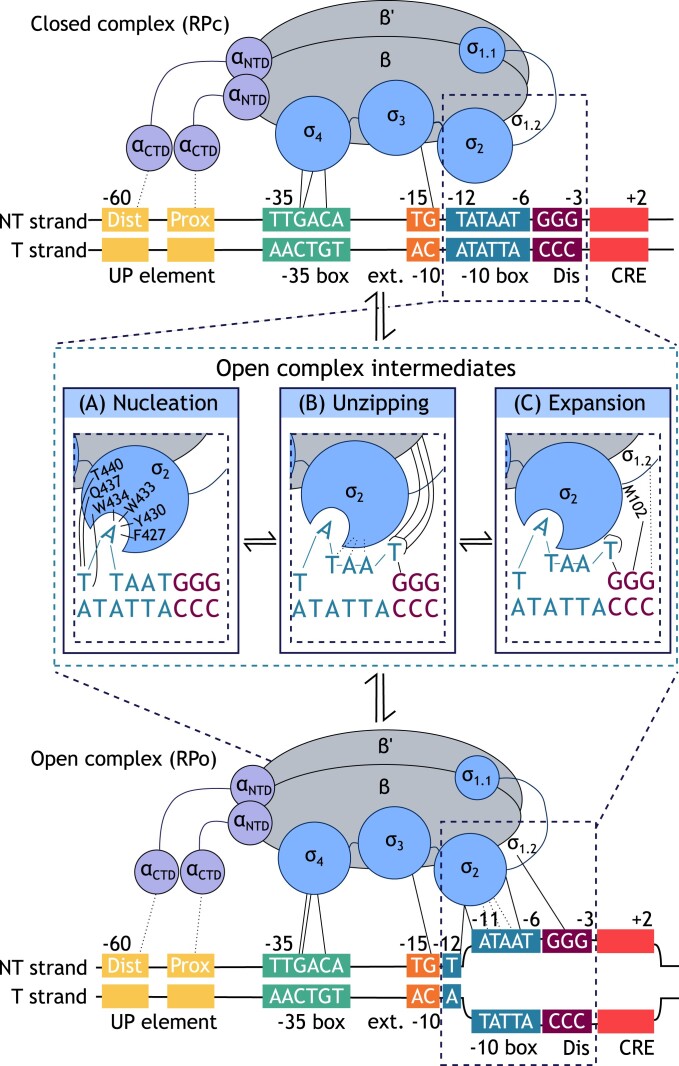
The proposed intermediates in the transition from closed to open complex. The interactions between RNAP holoenzyme (ααββ′ωσ) and 10 box promoter DNA that result in transcription bubble formation are shown. Solid lines indicate base-specific DNA contacts whilst broken lines show nonbase specific interactions of the holoenzyme with the DNA phosphate backbone. Both the template strand (T) and nontemplate strand (NT) of the promoter are given. The numbers given above each promoter element indicate distance from the transcription start site.

Once RNAP is recruited and anchored into position on the promoter DNA, transcription can proceed through unzipping of the DNA from positions −11 to +2, forming a transcription bubble, which is referred to as the DNA open-complex (RP_o_), a process summarized in Fig. 3. This strand separation is crucial to allow positioning of the transcription start site near the catalytic Mg^2+^ of the RNAP core enzyme active site, which runs between the β’ and β subunits, allowing the enzyme to catalyse initial RNA synthesis at the TSS (Zhang et al. 1999, Feklistov et al. 2017, Boyaci et al. 2019). This ability for the sigma factor to melt DNA is fundamental for functional transcription initiation, making the kinetics of open complex formation an important consideration in promoter engineering.

In recent structural and biochemical studies the mechanism of open complex formation at a number of promoters has been elucidated, and several reaction intermediates were identified (Ruff et al. 2015, Boyaci et al. 2019, Chen et al. 2020). Whilst it is now thought that specific −10 box features are not recognized by the open-clamp form of RNAP, which forms the closed-complex, subsequent transient clamp closure results in recognition of −10 box sequences. If favourable sequence features are present, this clamp closure results in nucleation of transcription bubble formation, starting the transition to RP_o_. If −10 box features are unfavourable for transcription initiation, the clamp reopens and continues scanning the genome for potential promoter sequences (Feklistov et al. 2017).

Nucleation of bubble formation involves initial separation of double-stranded DNA by flipping the nontemplate strand base at position −11 into a complementary pocket on the sigma factor. This step is dynamic and highly reversible, especially in the absence of interactions that stabilize the newly formed ds/ss junction (Fig. 3A). Following successful nucleation of the transcription bubble, the strands are further separated between positions −10 and −6, and single-stranded DNA of the template strand is pulled into the positively charged active site cleft. Stabilization of the strand-separated state can occur through flipping of the nontemplate nucleotide at position −7 into a second complementary pocket on the protein (Fig. 3B). Bubble expansion to position +2 and further stabilization of the strand-separated state results in an open complex that has the ability for functional transcription initiation (Fig. 3C).

Chen et al. (2021) have suggested that, although bacterial σ^70^ promoters most likely follow the same mechanism for open complex formation, the reaction kinetics and importance and longevity of different intermediates varies significantly with promoter sequence and RNAP composition (Ruff et al. 2015, Boyaci et al. 2019, Chen et al. 2021, Saecker et al. 2021). In addition, even at a given promoter, open complex kinetics may not be uniform and both stable and dynamic populations can exist. For example, at the lacCONS promoter, a particular open complex intermediate is formed with a frequency of 40%, whilst the remaining 60% of open complexes are formed without this intermediate (Malinen et al. 2022). The subsequent sections further outline the stages of open complex formation, detailing the interactions and important promoter features that are implicated in kinetics of formation and stability of the various intermediates.

#### −10 box

The sequence features of the −10 box are the primary determinant of open complex kinetics. Significantly, high AT content in the −10 box promotes strand separation as A−T bonds are known to require less energy to break than G−C bonds, explaining the AT rich consensus of the −10 box that is recognized by housekeeping sigma factors (Mo 2006, Khandelwal and Bhyravabhotla 2010). It is known that this alone can allow fast open complex kinetics. Whilst −11A and −7T are highly conserved, and flipping of these bases into complementary protein pockets contributes to fast initiation kinetics, their absence can be compensated for by a sufficiently AT rich −10 box, which works to lower the energy required to unzip the DNA, without the requirement for base-flipping (Heyduk and Heyduk 2014, Ruff et al. 2015). Equally, a recent thermodynamic model of RNAP–promoter interactions predicted that some GC rich hexamers also bind favourably to RNAP to activate transcription, indicating that it is not a requirement that the −10 box is AT rich (LaFleur et al. 2022). This flexibility allows housekeeping sigma factors to initiate transcription at diverse promoter sequences that are not a perfect match to consensus. This further highlights the complexity of the system by indicating that there is not one set formula of particular sequence features that need to be present. Conversely, interactions are additive to each other and should not be looked at independently when considering promoter strength and specificity.

At many σ^70^ promoters the primary interaction that nucleates initial transcription bubble formation is the flipping of the nontemplate strand base −11A out of the base stack and into a complementary protein pocket formed by the aromatic residues F427, Y430, W433, and W434 of σ_2.3_ (Panaghie et al. 2000, Lee, Lim and Adhya 2004, Feklistov and Darst 2009, 2011). This base-flipping nucleates strand separation and creates flexibility in the strand, allowing a 90° bend in the DNA, which is thought to direct single stranded downstream DNA towards the active site cleft (Feklistov and Darst 2011, Saecker et al. 2011, Ruff et al. 2015). It is currently unknown exactly what initiates base flipping but it has been suggested that transient closure of the RNAP clamp causes twisting of the DNA which facilitates −11A flipping into its complementary protein pocket (Ruff et al. 2015, Chen et al. 2020).

The importance of −11A base-flipping on open complex formation is demonstrated by a study in which alanine substitutions at the four amino acids that form the −11A specificity pocket prevented open complex formation, without having an effect on initial closed complex formation (Cook and DeHaseth 2007). This complements a previous study, which demonstrated that amino acid substitutions at Y430 and W433 caused defective nucleation of melting (Juang and Helmann 1994). Furthermore, when looking at the first 3 bp of the −10 box, the free energy model developed by LaFleur et al. (2022) showed that 10 out of 14 of the most favourable motifs, characterized by interaction free energies less than one, had an A at position −11, further indicating that this position is relatively conserved (LaFleur et al. 2022). However, despite this preference, there are numerous examples of functional σ^70^ promoters where the nucleotide at position −11 is not A, demonstrating that there is flexibility within this process. In such cases it is likely that stronger closed complex reactions can compensate for the lack of base flipping, creating the stability required to facilitate successful transcription (Koo et al. 2009).

Significantly, due to its high melting capacity, defined by presence of all four melting pocket residues, housekeeping sigma factors are more lenient to deviations from consensus promoter elements, whilst some alternative sigma factors require a near exact consensus sequence for successful promoter recognition (Helmann and Chamberlin 1988, Lonetto et al. 1992, Koo et al. 2009). This could be explained by the idea that base flipping by house-keeping sigma factors removes the requirement for strong closed complex interactions as it quickly stabilizes the initial open complex, preventing dissociation of the complex (Koo et al. 2009). All group 3 sigma factors lack one or more of the four critical melting residues that form the −11A pocket in housekeeping sigma factors (Feklístov et al. 2014). Mutating these residues back to the housekeeping consensus improved ability to melt DNA and relaxed the requirement for a perfect promoter, indicating that these amino acid residues can be targeted to alter promoter specificity. Where required, stringent promoter recognition could be achieved by mutating the relevant melting pocket residues, indicating how this information could be used for rational promoter design (Koo et al. 2009).

Interestingly, some alternative sigma factors have similar mechanisms for transcription bubble nucleation despite having very different consensus promoter motifs and more stringent promoter regulation. An example of this is the analogous ‘melting loop’ of σ^E^, which forms a complementary pocket for the base −11C, facilitating base flipping and subsequent transcription bubble nucleation in a comparable manner. Campagne et al. (2014) engineered the σ^E^ melting loop to recognize different bases at this position, showing that the composition of these amino acid residues directly affected promoter consensus. This demonstrates a powerful strategy for engineering promoters so that open complex nucleation is initiated specifically by its sigma factor pair with a complementary melting pocket.

The base at position −12T is highly conserved, indicating its importance for function (Feklistov and Darst 2011). This can be partially explained by the fact that certain combinations of nucleotide bases within a promoter are known to encourage base-unstacking, further facilitating base flipping and thereby influencing the reaction kinetics of bubble nucleation (Haugen et al. 2008, Ruff et al. 2015, Chen et al. 2021). Such stacking interactions dictate that the A nucleotide of a TA motif is the easiest base to flip, meaning that a T at position −12 is most thermodynamically favourable for flipping −11A into its complimentary protein pocket (Feklístov et al. 2014). Furthermore, this base pair at position −12 is directly, and sequence specifically, contacted on the template strand by Q437 and T440 of σ_2_, an interaction that remains during transcription bubble nucleation (Ruff et al. 2015). Mutation of these amino acid residues altered the preferred promoter consensus at the −12 position, implicating this position of the −10 box in sequence specific interactions with the sigma factor (Siegele et al. 1989, Waldburger et al. 1990). The involvement of these residues in interaction with position −12T was further confirmed by elucidation of the crystal structure of σ_2_ bound to −10 box DNA (Murakami et al. 2002). Despite the thermodynamic preference for T at position −12 and the fact that the top 4 most favourable −10 motifs, as predicted by the free energy model of LaFleur et al. (2022), all begin with TA, it is important to highlight that compositions with C, G, and A at position −12 also exhibit negative interaction energies. The fact that 9 out of 14 of the most favourable combinations, characterized by interaction free energies less than 1, do not involve −12T further emphasizes that the consensus at this position is not fixed (LaFleur et al. 2022).

Following transcription bubble nucleation, subsequent stabilization of the ds/ss junction occurs through a conformational change in amino acids W433 and W434, resulting in the formation of a wedge, which interacts with the exposed −12T base. Base flipping and subsequent conformational changes in W433/W434 are often dynamic, meaning that the structure of this early transcription bubble intermediate cannot always be elucidated (Chen et al. 2020). It has also been suggested that transient melting of −12T occurs, adding to the difficulty in elucidating the structure and reaction kinetics of the intermediate at this stage (Chen et al. 2020). To highlight their importance in stabilization of the ds/ss junction at σ^70^ promoters, mutation of W433 and W434 results in a highly deleterious phenotype, showing that these residues are essential for functional transcription of housekeeping genes (Park and Wang 2021). As an important enabler of open complex formation, interactions of the sigma factor with −12T could be targeted to redefine the selectivity or kinetics of transcription bubble nucleation and initial stabilization, in order to influence transcription rate and functionality.

In order to progress to a functional open complex, further bubble propagation and stabilization is required, which is also known to be sequence specific. During this process, bases of the nontemplate strand are bound through phosphate backbone interactions with sigma factor residues outside of the active site cleft, promoting strand separation. This is supported by early evidence from chemical crosslinks that show interactions between sigma factor residues and nontemplate strand DNA (Simpson 1979, Park et al. 1980, Hilton and Whiteley 1985, Buckle et al. 1991). In more stable open complexes these interactions are maximized and bases of the nontemplate strand (at positions −10 to −8) are stacked with each other (Saecker et al. 2021). Consequently, these interactions could be manipulated to influence open complex stability and kinetics.

To further stabilize the strand-separated state, single-stranded bases of the template strand at the −10 box position are pulled into the positively charged active site cleft, further stabilizing the strand-separated state. They are subsequently captured by electrostatic interactions with the inside of the cleft (Feklistov et al. 2017). A base-specific protrusion pocket on the sigma factor, which interacts with the template strand base at position −9 is thought to be specific for pyrimidines, meaning that this position has preference for a C or T on the template stand, which helps stabilize open complex formation when present (Chen et al. 2020). At most promoters, this is not rate limiting and, therefore, generally has little influence on kinetics of open complex formation. However, at weaker promoters this could be an important consideration, and therefore, a possible target for altering open complex stability.

Base −7T is highly conserved in promoters of both housekeeping and alternative sigma factors across the whole bacterial domain, implying its importance for function (Moyle et al. 1991, Feklistov and Darst 2011, Heyduk and Heyduk 2014). Its importance is further highlighted by the recent free energy model wherein all of the top 10 most favourable −10 box motifs all feature a T at position −7. Additionally, of the 23 −10 hexamers exhibiting negative free energies, indicative of favourable interaction energy, 16 featured −7T (LaFleur et al. 2022). Based on studies of open complex kinetics, it is thought that flipping of base −7T into a second complementary protein pocket on the sigma factor (formed by residues from σ_1.2_, σ_2.1_, and σ_2.3_) promotes further bubble propagation and stabilization, allowing the bubble to extend from position −11 to +2 (Feklistov and Darst 2011). Even when upstream bases are premelted by mis-match base pairing, a T to A mutation at this position prevented full bubble formation, suggesting that this base is involved in bubble propagation rather than nucleation of bubble formation (Chen et al. 2020). Aside from its role in bubble stabilization, flipping of −7T into its protein pocket is a prerequisite for displacement of σ_1.1_ from the active site cleft (Chen et al. 2020), providing a further explanation for the importance of this position in open complex formation and increasing its potential as a target in promoter engineering (Heyduk and Heyduk 2014, Ruff et al. 2015).

#### Discriminator element

The discriminator element spans from promoter positions −6 to −3 and is known to play a role in the kinetics and stability of the open complex. Interactions between the sigma factor and discriminator element positions −6 and −4 trap single stranded template DNA inside the active site channel, promoting separation of the strands and therefore further stabilizing the growing transcription bubble. Changes in the discriminator length and sequence are known to alter these interactions, leading to changes in the structure and stability of the RP_o_ and, therefore, influencing transcription rate (Saecker et al. 2021).

Studies on a number of promoters suggested that a GGG motif on the nontemplate strand between −6 and −4 gave the longest RP_o_ half-life. Of particular note in this study is the base −5G, which was shown to increase the half-life 10- to 50-fold at five different promoters, when compared to −5C (Haugen et al. 2006). In addition, it has been suggested that −6G can increase transcription rates at certain promoters if it is flipped into a complementary hydrophobic protein pocket on the surface of σ_1.2_, which stabilizes RP_o_ by preventing reannealing of the strands (Haugen et al. 2006, Zhang et al. 2012, Karpen and deHaseth 2015). Despite this proposed discriminator consensus, the recent free energy model published by LaFleur et al. (2022) shows that the 20 most favourable 3 bp discriminator motifs had comparable transcription initiation rates to each other, indicating that there is not one defined consensus sequence. Interestingly, many of the higher ranking motifs in terms of interaction free energy did not have a G at position −5 or −6 and the discriminator GGG had positive interaction free energy, indicating that, in the *in vitro* context in which this study was conducted, this motif was not particularly favourable for transcription initiation and suggesting that long RP_o_ half-life does not necessarily positively correlate with higher transcription rate (LaFleur et al. 2022).

The discriminator element interacts with σ_1.2_ to modulate these effects, with amino acid M102 making specific contacts with the nontemplate strand at position −5 (Haugen et al. 2006, 2008, Zhang et al. 2012, Basu et al. 2014, Zuo and Steitz 2015). Zhang et al. (2012) further confirmed this by showing that an alanine substitution at M102 decreases transcription rate by decreasing open complex lifetime. Interestingly, it is known that rRNA promoters often have a −5C consensus, as the resulting short open complex lifetime allows it to be subject to a lot of regulation (Travers 1990, Haugen et al. 2006). Thus, manipulating the discriminator element sequence of promoters with external regulators could be one way in which this knowledge could be used in promoter engineering. Although this is not a highly conserved promoter element, these interactions can help stabilize open complex formation, which is particularly significant at promoters with otherwise weak consensus motifs.

In addition to discriminator sequence, discriminator element length also plays a role in stability of the open complex. Research shows that the lifetime of the open complex decreases as discriminator element length increases (Jeong and Kang 1994, Liu and Turnbough Jr 1994, Lewis and Adhya 2004). Some of the loss of stability seen with longer discriminator elements is a result of a requirement for prescrunching of the DNA strands to produce an open complex with the designated TSS (Vvedenskaya et al. 2016).

#### Core recognition element

The core recognition element (CRE), located at position −4 to +2 relative to the TSS also has a role in stabilizing the open complex, allowing the transcription bubble to propagate to position +2. Interactions of RNAP core enzyme (β residues 84–642) with all nucleotides of the CRE were demonstrated in crosslinking experiments (Naryshkin et al. 2000). Importantly, +2G interacts sequence specifically with a pocket formed by β subunit residues R151, I445, D446, R451, L538, and V547, which further stabilizes the transcription bubble. Although not required for successful transcription initiation, the base G has a 5-fold lower RNAP off rate than any other base at this position, meaning that +2G makes the open complex more stable. Furthermore, nontemplate strand position +1 interacts with β amino acid W183, contributing to unstacking of +1 and +2 and consequently making the +2 base available for interacting with the CRE pocket (Zhang et al. 2012). As stable open complexes often lead to higher transcription rates, manipulating these stabilizing interactions could be a pathway for modulating transcription rate.

### Initial transcription and promoter escape

**Figure 4.**
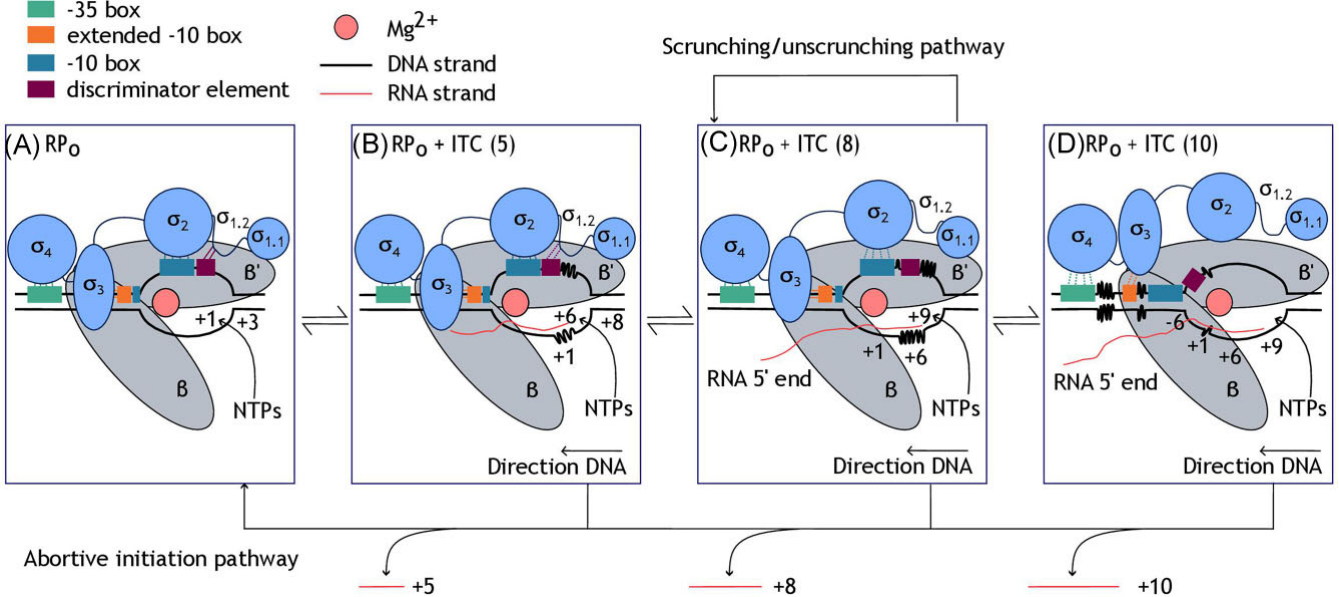
The mechanism of initial RNA synthesis and promoter escape in the RNAP active site cleft. The interactions between RNAP holoenzyme (ααββ’ωσ) and promoter are given at each stage of growth of the initial transcribing complex (ITC), culminating in promoter escape. Solid lines show RNAP–DNA interactions whilst disrupted interactions are represented by broken lines. Positions on the template strand are given, numbered according to distance from the transcription start site.

As the link between initiation and elongation, promoter escape defines transcriptional output at many promoters, and is therefore an important consideration for promoter prediction tools and promoter engineering (Reppas et al. 2006, Hatoum and Roberts 2008, Ko and Heyduk 2014). It should be noted that much of the information presented in this section relates to transcriptional regulation when NTP concentrations are low. When NTP concentrations are sufficiently high, promoter escape is not thought to be rate-limiting and many of the transcriptional regulation mechanisms that are described below are not observed (LaFleur et al. 2022). Due to the fact that cellular NTP levels can vary with environmental conditions, these mechanisms of promoter escape remain interesting to study when considering the predictability of biological systems in industrial conditions. The steps in initial RNA synthesis and subsequent promoter escape are shown in Fig. 4, which highlights the regulation involved in the transition to productive transcription.

When template strand DNA +1 and +2 positions are situated in the active site cleft, initial binding of nucleotide triphosphates (NTPs) at position +1 results in creation of the initial transcribing complex (ITC), an RNA–DNA hybrid to which further NTPs can be added. Subsequent translocation of the growing complex within the active site is essential to ensure that the template strand is in the correct position for further NTP binding and ITC elongation (Revyakin et al. 2006). Due to the fact that the RNAP holoenzyme remains held in position at promoter DNA by the interactions that are involved in open complex formation, the translocation of the short ITC within the enzyme active site cleft generates stress in the initiation complex as the upstream promoter DNA cannot move, resulting in scrunching of downstream DNA as the template strand moves further into the active site cleft, as illustrated in Fig. 4 (Kapanidis et al. 2006, Revyakin et al. 2006, Winkelman et al. 2015, Chen et al. 2021).

This stress build-up destabilizes RNAP–promoter contacts to drive promoter escape, and is relieved as RNAP–DNA contacts are broken (Henderson et al. 2019). A mechanism of downstream to upstream disruption of interactions has been proposed, starting with disruption of the interactions between RNAP and the discriminator and CRE elements, which are thought to be broken by translocation of an ITC containing a 5-mer RNA transcript into the active site (Fig. 4B). Disruption of the −10 box contacts is commonly the slowest step and results from translocation for synthesis of an ITC complex containing an RNA 9-mer (Fig. 4C). In the final stage of promoter escape, −35 box and upstream contacts are broken as a result of translocation for synthesis of 11-mer RNA transcript (Fig. 4D) (Henderson et al. 2017, Plaskon et al. 2021).

Whilst some of these growing ITCs escape the promoter (typically when the RNA transcript is 9–15 nucleotides in length), which allows them to transition to transcription elongation and go on to produce full length RNA transcripts, some complexes are nonproductive and release the small RNA (typically 2–10 nucleotides in length) from the hybrid as an alternative way to reduce the translocation stress (Heyduk and Heyduk 2018). This results in them returning to the open complex state in a process known as abortive initiation. These nonproductive complexes, which represent 30%–50% of open complexes under the reported conditions, can get stuck in cycles of abortive initiation and do not produce functional transcripts (Ko and Heyduk 2014, Duchi et al. 2016, Henderson et al. 2017, 2019).

Significantly, a considerable number of open complexes encounter transcriptional pause when ITC reaches a length of +6 as a result of σ_3.2_ occupying the RNA exit channel of the RNAP holoenzyme active site. This regulates continuation of transcription by creating a physical barrier that requires displacement before further extension of the RNA transcript can occur. To further demonstrate its influence, partial deletion of σ_3.2_ significantly reduced pausing at ITC +6 (Duchi et al. 2016). In one study of transcriptional pausing at the lac promoter, 80%–90% of transcripts were shown to enter this pause at ITC +6 and 20% of these did not exit the pause at the first attempt under the conditions tested (Dulin et al. 2018). As with the other examples of promoter escape mechanisms, the regulation of transcriptional pauses was demonstrated to be NTP concentration dependent. At low NTP concentrations the complexes were trapped in the paused state until NTP concentrations were sufficiently high (Dulin et al. 2018). Whilst 70%–80% of stalled constructs underwent abortive transcription, a second subset were thought to enter the scrunching–unscrunching pathway in which the RNA transcript was retained but transcription did not continue to the productive cycle (Dulin et al. 2018).

In this way, the productive pathway competes with the abortive pathway and scrunching pathway to regulate transcriptional output. These processes are subject to regulation that is, at least partly, influenced by the composition of the promoter sequence (Hsu et al. 2003, 2006, Chen et al. 2021, Saecker et al. 2021). The sections below outline the promoter elements involved in regulation of initial RNA synthesis and the kinetics of promoter escape.

#### Upstream promoter elements

The strength of RNAP–DNA interactions involved in formation of both the closed and open complex, as described above, have the biggest influence on the kinetics of promoter escape. Whilst it might be assumed that promoter sequences with a strong match to consensus motifs would result in a high transcriptional output, these sequences can be nonfunctional in low NTP conditions due to failure to disrupt the interaction between promoter and RNAP, resulting in an inability to move to further stages of transcription (Vo et al. 2003, Hsu et al. 2006, Ko and Heyduk 2014). To support this, it has been demonstrated that the highest gene expression levels *in vivo* were observed when either the −35 box or −10 box deviated from exact consensus (Henderson et al. 2019, Urtecho et al. 2019). This can be further explained by the fact that stable open complexes require more translocation stress and synthesis of a longer ITC to disrupt the initiation complex and allow promoter escape. As a result, stable open complexes often result in increased transcriptional pausing, higher yields of abortive products and lower promoter escape efficiency (Ellinger et al. 1994, Vo et al. 2003, Duchi et al. 2016, Saecker et al. 2021). As promoter escape can be the rate-limiting step at strong consensus promoters in low NTP conditions, it is an important target for promoter engineering (Einav and Phillips 2019).

Regulation of promoter escape is both sequence and position specific. The information in the previous sections relating to formation of a stable transcription bubble can be considered in this context, with the promoter elements that are mentioned this time having an inverse effect on productive transcriptional output. Consistent with its strong influence on open complex stability, changes in the −10 box sequence had the largest effect on promoter escape kinetics at low NTP concentrations, once again highlighting its importance (Ko and Heyduk 2014). However, it should be noted that these are not always inversely correlated. In one example, mutations at position −10 and −6 effected rate of promoter melting but did not influence the rate of promoter escape, showing that it is possible to effect one without changes in the other (Ko and Heyduk 2014).

Independently of influencing stability of interactions with the sigma factor, the sequence of the −10 box also has a role in influencing kinetics of initial transcription. In the RNA exit channel, steric and electrostatic interactions cause the 5-mer ITC to be pushed against σ_3.2_, resulting in contacts between the nontemplate −10 nucleotide and σ_2.3_, and template strand position −10 and σ_3_ (Zuo and Steitz 2015). These contacts are involved in displacement of σ_3.2_ in the exit channel, which is essential for further RNA synthesis and the progression of transcription. Thus, the bases of the −10 box influence both ability for and kinetics of the newly synthesized RNA proceeding through the RNA exit channel.

#### Discriminator element

The discriminator element also has a significant influence on the kinetics of initial RNA synthesis. Interaction between DNA template strand positions −4 and −3 with σ_3.2_ residues D514, D516, D517, and F522 helps to preorganize the template strand and force it to adopt an A-form helical conformation, which facilitates NTP addition by placing the strand in the correct position within the active site cleft (Zhang et al. 2012, Zuo and Steitz 2015). Mutations or deletions in amino acid residues that directly contact the DNA template strand impaired initial NTP binding (Pupov et al. 2014). Consistent with this, Henderson et al. (2017) show that a consensus discriminator element leads to longer abortive products and higher promoter escape efficiencies indicating that a strong discriminator prevents abortive initiation at early stages of initial transcription in low NTP conditions . Conversely, the presence of a Z-element like sequence at position −4 is thought to effect transcriptional output by interacting with the β′ subunit of RNAP during initial transcription, resulting in stabilization of σ factor-dependent RNAP pausing and increased abortive initiation from a synthetic promoter (Yuzenkova et al. 2011). These observations show that changes in the discriminator sequence can have an influence on promoter escape, and thus transcription rate. This is particularly significant in the case of strong consensus promoters, which could be matched with discriminator elements that favour promoter escape, in cases where high transcription rates are required.

#### Initially transcribed sequence

The composition of the initially transcribed sequence (ITS) from +1 to +20 can have a big influence on the outcome of transcription when NTP concentrations are low (Hsu et al. 2006, Davis et al. 2011, Heyduk and Heyduk 2018, Mazumder and Kapanidis 2019). Previous studies showed that changing the ITS effected promoter strength more than 10-fold (Kammerer et al. 1986, LaFleur et al. 2022). In particular, the ITS has been shown to influence the ratio of abortive to full length transcripts (Hsu et al. 2006). In general, a correlation between purine content of the ITS and high productive yield was observed, giving the first indication that this effect is sequence dependent (Hsu et al. 2006). Interestingly, whilst this influence is often independent of the sequence of the upstream promoter elements, certain upstream promoters have the ability to modulate ITS impact through allosteric effects (Heyduk and Heyduk 2018). This adds a further level of complexity and again highlights that elements should not be considered independently of each other, even as far as the downstream ITS. This means that the ITS should be considered according to its wider promoter context, implying that it should be defined as part of the promoter sequence.

One of the ways that ITS controls promoter escape and initial transcription is through regulation of transcriptional pausing. Base preferences within the ITS have been identified that match with known elongation pausing signals. The YG sequence motif is associated with long-lived initiation pauses as it is difficult for RNAP to transcribe, resulting in pausing in the context of other stresses such as that caused by σ_3.2_ blocking the RNA exit channel (Bauer et al. 2016, Dulin et al. 2018). The requirement for displacement of σ_3.2_ from the RNA exit channel induces a significant pause at most promoter sequences, making this a common point of regulation. A study on the lac promoter shows that replacing +6T and +7G on the nontemplate strand with +6G and +7T, resulted in a higher rate of initial transcription due to a shortening of the σ_3.2_-induced transcriptional pause (Dulin et al. 2018). Additional studies show that a range of other combinations at these positions also resulted in reduced transcriptional pausing (Bauer et al. 2016, Duchi et al. 2016). It is thought that amino acid D446 of the β-subunit of RNAP directly contacts ITS position +7G, influencing the transcriptional pause. Very few transcripts were able to extend beyond six nucleotides with an alanine substitution at D446 (Dulin et al. 2018). This preference was promoter specific, again indicating that upstream promoter sequence has a significant effect on the influence of the ITS. At low NTP concentrations, YG repeats at any position of the ITS effected the ability for promoter escape when in combination with stresses such as DNA scrunching or expansion of the transcription bubble (Dulin et al. 2018). This is because ITC translocation is unfavourable and rapidly reversible in the absence of the correct NTP, due to the resulting stress build-up, making the kinetics of promoter escape strongly dependent on NTP concentration (Dulin et al. 2018).

ITS also regulates promoter escape through interactions that contribute to stability of the growing DNA–RNA duplex. High stability in the first 10 bps, which is defined by the composition of the ITS, correlated with fast promoter escape. This can be explained by the fact that high stability reduces the chance of disassociation or misalignment of the transcript within the active site (Heyduk and Heyduk 2018). Furthermore, the structure of the RNA 5′ end, which is also defined by the ITS, has a role to play in kinetics of promoter escape through its interaction with σ_3.2_. Charge repulsion between the growing RNA and σ_3.2_ are involved in displacing σ_3.2_ from the active site cleft to allow elongation of the initially transcribed complex (Basu et al. 2014, Pupov et al. 2014). To further support this, studies show that the structure of the 5′ RNA end controls rate of productive pause exit when ITC reaches +6, which is known to be the point at which σ_3.2_ is displaced from the RNA exit channel (Dulin et al. 2018). Whilst 5′ RNA structure is influential, amino acid substitutions in σ_3.2_ did not significantly affect these interactions, indicating that base-pair specific interactions with specific amino acids of σ_3.2_ are not involved in this regulation (Pupov et al. 2014).

## Conclusion

The knowledge presented in this review, relating to the influence of promoter sequence on the mechanisms and kinetics of transcription initiation, can be utilized in the development of a more rational approach to promoter engineering. This knowledge-based approach can help us to design more predictable, tuneable, and orthogonal promoter sequences, which is important in the context of regulation of gene expression.

A highly significant take-home message is that transcription initiation is complex and that underestimating this prevents the predictability of promoter engineering. Whilst the transcription mechanism is largely conserved, the kinetics of transcription initiation are strongly promoter sequence dependent. Promoter activity, strength, and specificity is a function, not just of DNA recognition and binding, but also of DNA melting and promoter escape, meaning that the stages of transcription initiation cannot be considered independently of each other. For example, a balance in RP_o_ stability is required for functional transcription as stable open complexes are often limited at promoter escape.

An interplay of different promoter characteristics and their interaction with the sigma subunit of the RNAP holoenzyme determines the resulting transcriptional output. Despite the fact that each promoter region has a specific role in recognition, melting, and initial transcription, these interactions are additive to each other and sit within the more general context of the promoter sequence, structure, and surrounding regulation, meaning that individual promoter motifs cannot be looked at independently when defining promoter strength and specificity. Building on suggestions from previous reviews, different classifications of promoter sequence could be foreseen that categorize promoters based on the combination of promoter elements they contain (Hook-Barnard and Hinton 2007). Such classification could be used to define the promoter engineering strategy. For example, it is known that promoters with weak −35 and −10 consensus motifs show high dependence on other promoter features such as consensus Z element. This could direct the interactions that should be targeted to tune promoter strength or specificity at a given promoter sequence.

Of equal importance is that the knowledge gathered here shows that it is essential that upstream and downstream regions are defined as part of the promoter sequence, as they often have a significant influence on promoter output. This suggestion is supported by several studies such as that by Davis et al. (2011) who characterized a set of promoter sequences that include a downstream insulator following the observation that the 20–30 nucleotides following the transcription start sites can also have a significant impact on promoter clearance, and thus transcription initiation rate (Davis et al. 2011, Balzer Le et al. 2020). Urtecho et al. (2019) studied the relationships between promoter elements by combining different elements across different promoter backgrounds. It was shown that, whilst 74% of variation resulted from changes in the −35 and −10 boxes, 19% of variation could not be explained by linear relationships between elements and likely resulted from more complex relationships between the elements, further confirming the complexity of promoter regulation. However, these combinations focus on upstream promoter elements and are still limited in the number of different combinations tested, meaning that the complexity is likely still underestimated.

Promoter prediction tools are already being improved to take such observations into account. A number of recent models have provided significant insight into the relative contributions of different promoter motifs on controlling transcription rate, providing a valuable resource for studying the influence of promoter sequence on transcription initiation rate (Einav and Phillips 2019, Urtecho et al. 2019, Lagator et al. 2020, LaFleur et al. 2022). The Salis promoter calculator, which is based on a thermodynamic model of RNAP–DNA interactions, already considers a wide range of promoter elements in its training dataset, and generates quantitative predictions on the favourability of different compositions of both upstream and downstream elements. Such datasets can be utilized for forward engineering of promoter sequences, providing the opportunity to create promoters with desired expression properties, or to remove sequences in the surrounding DNA that might interfere with transcription, therefore, giving an example how an in-depth understanding and quantitative data on the interactions involved in transcriptional control can be used to rationally design new promoter sequences (LaFleur et al. 2022). In a further example of how a data driven approach can be utilized in promoter engineering, Wang and colleagues combine knowledge-based promoter design with AI based generation of promoter sequences to significantly improve promoter performance, further highlighting this potential (Zhang et al. 2023).

Whilst there is already a wealth of knowledge that can be utilized in the context of promoter engineering, the complexity of transcription initiation remains a limiting factor. More detailed mechanisms of RP_o_ formation and the associated reaction kinetics continue to be elucidated and the dynamic nature of transcription initiation means that, at certain positions of unstable intermediates, the structure is not fully resolved. Furthermore, many of these structural models of transcription initiation have been conducted on a limited number of promoters and under one set of environmental conditions, limiting their broad applicability.

It is known that the mechanism of transcription initiation, and especially the reaction kinetics, are strongly dependent on both the promoter sequence and the environmental context. Significantly, under different environmental conditions certain promoter features become more or less influential, meaning that the importance of different promoter motifs on transcriptional output varies with wider promoter sequence and environmental context. Whilst recent quantitative models of transcription rate allow increasingly accurate predictions of promoter strength, the complexity of the system, and this context dependence, means that there are still some significant limitations to our knowledge of transcription initiation. Nonetheless, recent rapid advancements in the field, coupled with high throughput screening techniques and greater data processing capabilities, suggest that these gaps in our knowledge can be quickly filled, enhancing our ability to reliably predict and forward engineer improved promoter sequences for use in a variety of applications.
